# Positive regulation of Type III secretion effectors and virulence by RyhB paralogs in *Salmonella enterica* serovar Enteritidis

**DOI:** 10.1186/s13567-021-00915-z

**Published:** 2021-03-10

**Authors:** Binjie Chen, Xianchen Meng, Jie Ni, Mengping He, Yanfei Chen, Pengpeng Xia, Heng Wang, Siguo Liu, Guoqiang Zhu, Xia Meng

**Affiliations:** 1 College of Veterinary Medicine, Yangzhou University, Yangzhou, 225009 China; 2 Jiangsu Co-Innovation Center for Prevention and Control of Important Animal Infectious Diseases and Zoonoses, Joint International Research Laboratory of Agriculture and Agri-Product Safety of Ministry of Education of China, Yangzhou, 225009 China; 3 Division of Bacterial Diseases, State Key Laboratory of Veterinary Biotechnology, Harbin Veterinary Research Institute, Chinese Academy of Agricultural Sciences, Harbin, Heilongjiang China

**Keywords:** RyhB, Regulation of virulence, SipA, Invasion, Pathogenicity, *Salmonella*

## Abstract

Small non-coding RNA RyhB is a key regulator of iron homeostasis in bacteria by sensing iron availability in the environment. Although RyhB is known to influence bacterial virulence by interacting with iron metabolism related regulators, its interaction with virulence genes, especially the Type III secretion system (T3SS), has not been reported. Here, we demonstrate that two RyhB paralogs of *Salmonella enterica* serovar Enteritidis upregulate Type III secretion system (T3SS) effectors, and consequently affect *Salmonella* invasion into intestinal epithelial cells. Specifically, we found that RyhB-1 modulate *Salmonella* response to stress condition of iron deficiency and hypoxia, and stress in simulated intestinal environment (SIE). Under SIE culture conditions, both RyhB-1 and RyhB-2 are drastically induced and directly upregulate the expression of T3SS effector gene *sipA* by interacting with its 5′ untranslated region (5′ UTR) via an incomplete base-pairing mechanism. In addition, the RyhB paralogs upregulate the expression of T3SS effector gene *sopE*. By regulating the invasion-related genes, RyhBs in turn affect the ability of *S.* Enteritidis to adhere to and invade into intestinal epithelial cells. Our findings provide evidence that RyhBs function as critical virulence factors by directly regulating virulence-related gene expression. Thus, inhibition of RyhBs may be a potential strategy to attenuate *Salmonella*.

**Supplementary Information:**

The online version contains supplementary material available at 10.1186/s13567-021-00915-z.

## Introduction

*Salmonella* is an important zoonotic pathogen that causes foodborne enteritis, which is frequently reported worldwide. According to the estimate by Centers for Disease Control and Prevention (CDC), ~ 1.2 million people in the United States are affected by foodborne salmonellosis each year, resulting in about 450 deaths and 23 000 hospitalizations [1]. Salmonellosis is the second most commonly reported gastrointestinal infection in the European Union where it is mainly caused by *Salmonella enterica* serovar Enteritidis (SE) and *Salmonella enterica* serovar Typhimurium [2]. In China, *Salmonella* is a widely distributed pathogen and is responsible for substantial socioeconomic burden. According to a recent report, the isolation rate of *Salmonella* species from diarrheal patients in China was 2.9% during 2013–2016, and the top serotypes identified in the study were *S*. Typhimurium and *S*. Enteritidis [3]. SE has become the major cause of foodborne salmonellosis worldwide and is mainly transmitted through contaminated poultry products and eggs [4, 5].

During the process of infecting the intestine, *Salmonella* penetrates the mucosal layer and is internalized into the intestinal epithelium. This step is directed by the delivery of a series of effector proteins into host cells via the Type III secretion systems (T3SS) that are encoded by *Salmonella* pathogenicity island 1 (SPI-1) and SPI-2 [6, 7]. Over 30 T3SS effectors collaboratively manipulate the host cell cytoskeleton, membrane trafficking, signal transduction, and pro-inflammatory responses [7]. T3SS effectors SipA and SopE play a crucial role in *Salmonella* invasion of intestinal epithelial cells. SipA drives actin polymerization, thereby resulting in the rearrangement of actin cytoskeleton (membrane ruffling) and internalization of bacteria by intestinal epithelial cells [7, 8]. During infection, SipA cooperates with an SPI2 effector SifA to ensure perinuclear *Salmonella*-containing vacuole (SCV) positioning and regulate SCV maturation and bacterial intracellular replication [9]. In macrophages, SipA activates caspase-3 and mediates survival at the early stages of *S.* Typhimurium infection [10]. SopE activates host Rho GTPases by catalysing the exchange of GDP for GTP and indirectly modulates host actin cytoskeleton [11]. SopE cooperates with host ADP ribosylation factor 1 (Arf1) activator (ARNO) to trigger *Salmonella* invasion [12]. Both SipA and SopE can disrupt tight junctions that likely lead to polymorphonuclear leukocyte (PMN) transmigration [8, 13]. It is known that T3SS effector production is modulated by transcriptional factors (e.g. HilA, HilD and InvF), the ArcAB global regulatory system, and post-transcriptional regulators AraC/XylS [14, 15]. However, the regulation of T3SS effector production by non-coding small RNA has not yet been reported.

Non-coding small RNAs (sRNAs) are a class of RNA molecules of 40–500 bp in length that are located within intergenic regions and transcribed in the genome but do not encode protein [16]. sRNAs usually modulate the translation or stability of target mRNAs by binding to the 5′ or 3′ untranslated region (UTR) of target mRNAs through incomplete base pairing [17]. Some sRNAs regulate target mRNA translation by pairing with the 5′ coding region [18]. One sRNA may regulate multiple target mRNAs, while one target mRNA may be regulated by multiple sRNAs [17]. sRNA RyhB was first identified in *Escherichia coli* [19], and soon afterward, RyhB and its homologs were found in several bacterial species such as *Salmonella enterica*, *Yersinia pestis*, *Pseudomonas aeruginosa*, *Vibrio cholera*, and *Shigella dysenteriae* [20–22]. Interestingly, *E. coli* only encodes one RyhB sRNA, whereas *Salmonella* encodes two RyhB paralogs, namely, RyhB-1 and RyhB-2, which have high sequence homology to *E. coli* RyhB [21]. Although the positions of RyhB-1 and RyhB-2 in *Salmonella* are far apart in the genome, their nucleotide sequence homology is relatively high (55.3%). In addition, they have a highly conserved 33-bp regions [21]. RyhB paralogs in *Salmonella* act singularly or together in modulating target gene expression by sensing changes in the external environment, which in turn affects a variety of physiological processes in *Salmonella*, including adaptive response to oxidative stress, iron metabolism, nitrate homeostasis, chemotaxis, and intracellular survival [23–26]. Although RyhB paralogs have been extensively studied in *S.* Typhimurium, little has been done in *S*. Enteritidis, and information is particularly scarce regarding the regulation of virulence-related genes by RyhB paralogs. To date, only a few virulence and motility-related genes (*flgJ* and *fliC*) have been found to be regulated by RyhBs in *Salmonella* or *Escherichia coli* [24, 27]. Although previous studies have demonstrated that RyhBs play critical roles in maintaining *Salmonella* viability in eukaryotic epithelial cells and survival in macrophages [28, 29], how they contribute to these phenotypes remains to be elucidated.

Here, we present a study on the function of RyhB paralogs in modulating *S*. Enteritidis virulence. We identified target genes of RyhB paralogs via a simulated intestinal environment (SIE) model in vitro and demonstrated that both RyhBs upregulated the expression of *Salmonella* T3SS effector genes *sipA* and *sopE*, thereby affecting the ability of *Salmonella* Enteritidis to invade intestinal epithelial cells.

## Materials and methods

### Bacteria, plasmids and cell growth conditions

Bacterial strains and plasmids used throughout this study are listed in Table 1. All *Salmonella enterica* serovar Enteritidis mutants were derived from the WT strain CMCC (B) 50336. All bacteria were grown routinely in Luria–Bertani (LB) broth or on LB plates at 37 °C, except for mutants containing the temperature-sensitive plasmids pCP20 or pKD46, which was grown at 30 °C. Bacteria harbouring antibiotic resistance genes were cultured in LB containing ampicillin (Amp, 100 μg/mL) (Sangon Biotech, Shanghai, China), kanamycin (Kan, 50 μg/mL) (Sangon Biotech), or chloramphenicol (Cm, 34 μg/mL) (Sangon Biotech) when appropriate. Aerobic growth was conducted in a bacterial shaker at 180 rpm. Anaerobic growth was achieved by static culture at 37 °C in the anaerobic workstation (DG250, Don Whitley Scientific, UK) with mixed gas (10% H_2_, 10% CO_2_, and 80% N_2_). The iron chelator 0.2 mM 2, 2′-dipyridyl (Solarbio, Beijing, China) was added to the LB medium for iron-limited growth. To mimic the intestinal environment, growth was performed by culturing *Salmonella* Enteritidis in a simulated intestinal fluid medium (pH 6.8), which contained 0.05 mol/L KH_2_PO_4_ and 10 g/L trypsin at 37 °C in an anaerobic workstation [30–32]. To determine growth rates, bacteria were incubated at 37 °C with continuous agitation (180 rpm) in LB broth overnight. Then, the bacteria cultures were transferred to LB broth, iron-limited LB broth, and simulated intestinal fluid medium respectively at a ratio of 1:100 and cultured in aerobic or anaerobic conditions. The number of live bacteria was measured once every two hours by counting colonies on LB plates. *E. coli* DH5α and TOP10 cells were used as hosts for plasmid DNA manipulation and protein production, respectively. Human colorectal adenocarcinoma epithelial cells (Caco-2) were cultured as described elsewhere [33].

**Table 1:** Bacteria and plasmids used in this study

Strain/plasmids	Characteristics	References
Strain		
CMCC(B)50336	*Salmonella enterica* serovar Enteritidis wild-type	NICPBP, China
△*ryhB-1*mutant	*ryhB-1* deficient mutant	This study
△*ryhB-2* mutant	*ryhB-2* deficient mutant	This study
△*ryhB-1*/△*ryhB-2* mutant △*ryhB-1*/p*ryhB-1* mutant △*ryhB-2*/p*ryhB-2* mutant △*ryhB-1/*△*ryhB-2*/*pryhB-1*/*pryhB-2* mutant	*ryhB-1* and *ryhB-2* deficient mutant △*ryhB-1* carrying pBR-*ryhB-1* (Amp^r^) △*ryhB-2* carrying pACYC-*ryhB-2* (Cm^r^) △*ryhB-1/*△*ryhB-2* carrying pBR-*ryhB-1* and pACYC-*ryhB-2*	This study This study This study This study
Plasmids		
pKD3	Cm^r^; Cm cassette template	[34]
pKD46	Amp^r^; λ-red recombinase expression	[34]
pCP20	Amp^r^, Cm^r^; Flp recombinase expression	[34]
pGEM-T easy	Amp^r^; cloning vector	Takara
pMD19 T-simple pBR-*ryhB-1* pACYC-*ryhB-2*	Amp^r^; cloning vector pBR322 carrying the entire *ryhB-1* nucleotide sequence pACYC184 carrying the entire *ryhB-2* nucleotide sequence	Takara This study This study
pJV-300	Amp^r^; sRNA cloning vector	[40]
pXG-0	Cm^r^; negative control vector without GFP	[40]
pXG-10SF	Cm^r^; target gene cloning vector with GFP	[40]

### Construction of the deletion mutants and the complemented mutants

All primers used for RyhBs gene cloning and mutant construction in this study are given in Additional file 1. The sequences of *ryhB-1* and *ryhB-2* in SE50336 were amplified using primer pairs “*vryhB-1*-F, *vryhB-1*-R” and “*vryhB-2*-F *vryhB-2*-R” by PCR, respectively. After sequencing the PCR products, the sequences of *ryhB-1* and *ryhB-2* were blasted against RyhB sequences in *S*. Typhimurium. The *ryhB* homologs deletion mutants were constructed using the λ-Red mediated recombination system as previously described [34, 35]. Briefly, primer pairs *ryhB-1*-F and *ryhB-1*-R, as well as *ryhB-2*-F and *ryhB-2*-R were used to amplify the chloramphenicol resistance (Cm^r^) cassette from plasmid pKD3, including 48-bp homology extensions from the 5′ and 3′ of the *ryhB-1* and *ryhB-2* nucleotide sequences. The PCR products were purified and introduced into plasmid pKD46-containing *S*. Enteritidis 50336 to obtain the Cm^r^ recombinant bacteria, and then the Cm cassette was removed using plasmid pCP20. The *ryhB-1* and *ryhB-2* complete deletion mutants △*ryhB-1* and △*ryhB-2* were obtained and confirmed by PCR screening using primers (*vryhB-1*-F and *vryhB-1*-R, *vryhB-2*-F and *vryhB-2*-R) and DNA sequencing. The double deletion mutant △*ryhB-1/*△*ryhB-2* was generated by operating the *ryhB-1* deletion process in mutant △*ryhB-2*. The complemented strain was generated by cloning the full-length *ryhB* sequence into plasmid pBR322 or pACYC184, which was transformed to the corresponding *ryhB* mutant.

### Quantitative real-time PCR

*S*. Enteritidis 50336 and RyhB mutants were grown in simulated intestinal fluid medium for 2 h at 37 °C in an anaerobic workstation and collected by centrifugation. Total RNA was extracted using TRIzol reagent (Invitrogen, CA, USA). cDNA was synthesized using the PrimeScript RRT reagent kit with gDNA Eraser (Takara, Tokyo, Japan). Relative transcript abundance was determined using RT-qPCR with SYBR Premix Ex Taq II (Takara) and the primers listed in Additional file 2 using an ABI7500 instrument (Applied Biosystems, USA). Assays were performed in triplicate, and all data were normalized to the endogenous reference gene *gyrA* using the 2^−△△^CT method.

### Prediction of interactive sites and the secondary structure of target mRNA

RyhB-1, RyhB-2, and their candidate target gene sequences were submitted to the IntaRNA website to predict their interaction sites [36–38]. According to the website recommendation, the mRNA sequence should comprise 150-nt UTRs upstream of the start codon of a candidate gene and 150-nt coding sequence starting from the start codon. If the UTR is less than 150-nt in size, all UTR sequences should be used for prediction. Based on the recommended parameters by the website, a 168-nucleotide sequence, which contained the entire 5′ UTR sequence of *sipA* (18 bases) and the first 150 bases of the *sipA* coding sequence, as well as sequences of *ryhB-1* and *ryhB-2* were submitted to the website for *sipA-ryhB* interaction site prediction. For *sopE-ryhB* interaction site prediction, a 300-nt sequence that contained a 150-nt 5′ UTR of the *sopE* and the first 150-nt coding sequence of *sopE*, as well as sequences of *ryhB-1* and *ryhB-2* were submitted to the website. The secondary structure of a candidate mRNA was predicted by the RNAstructure module of CLC Main Workbench (5.5) [39].

### A GFP fusion approach to validate interactions between RyhB and targets

The sRNA-target interaction was detected using the GFP-based reporter system as previously described [40]. Briefly, *E. coli* strain Top10 was used to clone GFP fusions and in all experiments that involved co-expression of GFP fusions and sRNAs. pXG-10SF plasmid (carried the chloramphenicol resistance cassette) was used to clone target-GFP fusion. pXG-0 (carried the chloramphenicol resistance cassette) was used as control plasmid to determine cellular autofluorescence. pJV-300 plasmid (carried the ampicillin resistance cassette) was used to clone sRNA RyhB-1 and RyhB-2. All the above plasmids were provided by Prof. Zhengfei Liu from Huazhong Agricultural University in China. Growth in LB broth or on LB plates at 37 °C was used throughout this study. Antibiotics were applied at the following concentrations: 100 μg/mL ampicillin, and 34 μg/mL chloramphenicol. The complete list of oligonucleotides used for cloning is provided in Additional file 3. The 5′ UTR sequence of *sipA* was cloned upstream of *gfp* gene’s initiation codon in the GFP expression plasmid pXG-10SF by the sequence and ligation-independent cloning (SLIC) technology [41] to construct fusion plasmid 5′ UTR *sipA*-pXG-10SF. The *ryhB-1* and *ryhB-2* sequences were separately cloned into sRNA expression plasmid pJV-300 by the SLIC technology to generate plasmids *ryhB-1*-pJV-300 and *ryhB-2*-pJV-300, respectively. Fluorescence of *E. coli* TOP10 harbouring the *gfp* fusion plasmid and sRNA expression plasmid (named as *ryhB-1*::5′ UTR *sipA*-*gfp* and *ryhB-2*::5′ UTR *sipA*-*gfp* separately) was compared with that of *E. coli* TOP10 harbouring *gfp* fusion plasmid and pJV-300 (named as “no sRNA::5′ UTR *sipA*-*gfp*”) in both whole-cell colony plate and whole-cell liquid culture assays to determine the interaction between RyhB and *sipA*. The same method was also used to construct *sopE* related expression vector.

To measure whole-cell colony fluorescence on plate, the above constructed strains were cultured to log phase and diluted 10^6^ times, and then the diluent was dropped onto a LB plate containing 100 μg/mL Amp and 34 μg/mL Cm and incubated at 37 °C for 16 h to obtain smear of colonies. The morphology and fluorescence intensity of single colonies were observed and imaged using the natural light mode and the fluorescence mode, respectively, by using an inverted fluorescence microscope (Carl Zeiss, Jena, Germany). To measure whole-cell fluorescence in liquid culture, *E. coli* TOP10 harbouring a *gfp* fusion plasmid and a sRNA expression plasmid was cultured in LB liquid medium at 37 ℃ with shaking at 200 rpm to OD600 2.0. All the cultures were washed three times and resuspended with equal volume PBS. For each culture, 150 μL cell suspension was taken and transferred to a 96-well microtiter plate (Corning, NY, USA) and fluorescence was measured at 37 ℃ (optical excitation filter 485/20 nm, emission filter 528/20 nm, tungsten lamp energy, measurement height 8.0 mm) using a Microporous plate detector (Synergy 2) (Biotek, Vermont, USA). To calculate absolute fluorescence of a given strain, the mean fluorescence of the three aliquots from three independently grown cultures was determined. PBS was used as blank control and *E. coli* TOP10 strains harbouring pXG-0 and pJV-300 plasmid were used as negative control (cellular autofluorescence). The absolute fluorescence of a given strain harbouring a *gfp* fusion plasmid and a sRNA expression plasmid was its measured fluorescence units subtracted by the fluorescence sum of blank control and negative control.

Besides, the whole-cell proteins of strains expressing fluorescent GFP fusions were analysed by western blotting to verify the expression level of GFP. The whole-cell protein fractions were prepared as described previously [40]. Briefly, 20 μg of whole-cell protein fraction of each strain were separated by 15% SDS–PAGE. Gels were blotted onto PVDF membrane using a Trans-Blot SD system (Bio-Rad, CA, USA). Membranes were rinsed in TBST20 buffer (20 mM Tris base, 150 mM NaCl and 0.1% Tween 20), blocked for 3 h in10% skimmed milk, and incubated with a GFP monoclonal antibody (Proteintech, Chicago, USA) for 2 h at room temperature. The blots were washed three times (10 min each) in TBST20. Then the membranes were incubated with a-goat-horseradish peroxidase (HRP) anti-mouse IgG (Proteintech) (1:5000 in 10% skimmed milk in TBST20) for 1 h at room temperature. The blots were washed three times in TBST20 and developed using a NcmECL Ultra Kit (Ncmbio, Suzhou, China). To ensure an equal amount of protein loading in different samples, the same membranes used for GFP blotting were regenerated and then re-blotted with antibodies against glyceraldehyde-3-phosphate dehydrogenase (GAPDH) (Proteintech) using the same protocol.

### Bacterial adherence and invasion assays

Caco-2 cells were cultivated in Dulbecco’s minimal Eagle medium (DMEM) (Gibco, NY, USA) containing glutamine (Gibco) supplemented with 10% heat-inactivated fetal bovine serum (FBS) (Gibco). Cells were maintained in an atmosphere of 5% CO_2_ at 37 °C.

Bacterial adherence and invasion into Caco-2 cells were performed as previously described [35]. Bacteria were incubated on a monolayer of 1 × 10^5^ Caco-2 cells at a multiplicity of infection (MOI) of 100 at 37 °C in 96-well tissue culture plates (Corning, NY, USA) for 2 h. Infections were performed in triplicate. For adhesion assay, infected cell monolayers were gently washed three times with PBS to remove loosely adherent bacteria. Cells were lysed with 0.5% Triton X-100 (Solarbio) for 30 min. The lysates were serially diluted and plated onto LB agar plates for the enumeration of adherent bacteria. For invasion assay, infected cell monolayers were gently washed three times with PBS to remove non-adherent bacteria after 2 h of infection. Then 200 μL PBS with 100 μg/mL gentamicin was added to each well and incubated at 37 °C for 1 h to kill bacteria that adhere to the cell surface. After 1 h incubation, cells were washed gently with PBS and lysed with 0.5% Triton X-100 for 30 min. The lysates were serially diluted and plated onto LB agar plates for counting invaded bacteria.

Bacterial adherence and invasion in a Murine Ex vivo Anaerobic Tissue (MEAT) model were performed as previously described [30, 31]. Bacteria were grown in LB to early stationary phase (18 h), harvested by centrifugation and diluted in PBS prior to invasion assay. SPF-grade Balb/C mice (5-week-old) were fed for two days in advance with sterile water containing 100 μg/mL streptomycin to remove intestinal bacteria. The mouse small intestines were cut open, dissected to several about 2-cm pieces, and mounted in dishes covered with DMEM medium. Approximately 10^11^ CFU WT or *ryhB* mutants were loaded on the opened intestinal tissues, and dishes were placed in an anaerobic workstation at 37 °C for 3 h. The exact number of bacteria in an inoculum was determined by CFU counts on LB plate. PBS was incubated on the surface of intestinal tissues as a negative control. After 3 h inoculation, the tissue was gently washed thrice with PBS to remove loosely adherent bacteria and then lysed with tissue mill. The lysates were serially diluted and plated onto Salmonella-Shigella (S.S.) agar plates (Solarbio) for the enumeration of adherent and invaded bacteria. Meanwhile, the bacteria in lysates were also identified by amplifying *Salmonella* specific gene *sdf1* by PCR [42] for reliable *Salmonella* enumeration. The animal experiments followed the National Institute of Health guidelines for the ethical use of animals in China. All procedures were approved by the Animal Care and Ethics Committee of Yangzhou University (permit number: YZUSYXY2019-0032).

### Statistical analysis

The data of bacteria growth and fluorescence measurements were analyzed using unpaired Student’s *t*-test for independent samples by GraphPad Prism software (GraphPad Software, San Diego, California, USA). Differences were considered significant when *p* ≤ 0.05. The data of qRT-PCR, adhesion and invasion assays were analyzed with SPSS 17.0 software (SPSS, Chicago, USA) using one-way ANOVA for multiple comparisons.

## Results

### Characteristics of *ryhB-1* and *ryhB-2* and their RNA secondary structure in *S*. Enteritidis

Based on the sequences of the *ryhB* paralogs (*ryhB-1* and *ryhB-2*) in *S.* Typhimurium LT2 [21], we cloned and analysed the *ryhB-1* and *ryhB-2* genes in *S*. Enteritidis strain CMCC (B) 50336 (abbreviated as SE50336). The results showed that *ryhB-1* and *ryhB-2* (GenBank accession numbers: MW583716 and MW583717) in SE50336, shared 99% and 100% identity to the two *ryhB* paralogs in S. Typhimurium LT2, respectively. This result is concordant with a previous study on highly conserved *ryhB* in all sequenced genomes of *Salmonella* [21]. BLAST analysis of *ryhB-1* and *ryhB-2* showed that the two genes in SE50336 shared 65% identity.

**Figure 1:**
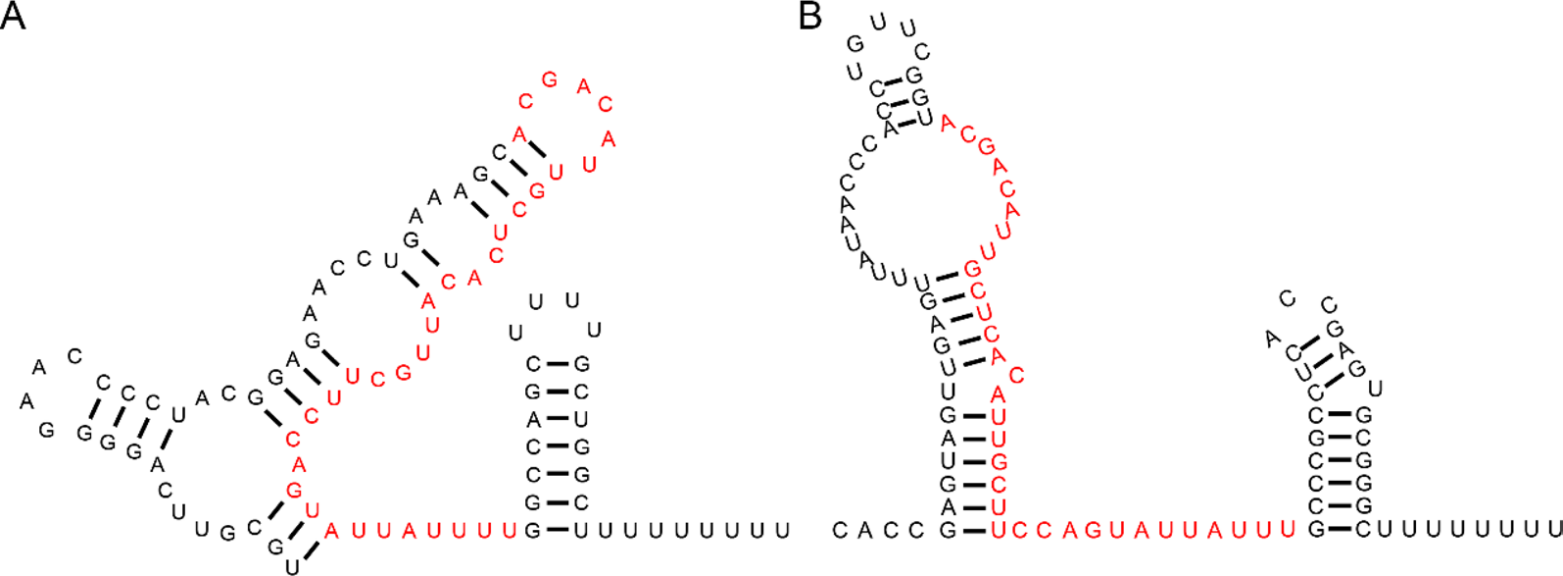
**The secondary structure of RyhB-1 (A) and RyhB-2 (B) in S. Enteritidis as predicted by RNAstructure module of CLC Main Workbench (5.5) software**. The 33-bp homologous sequence is marked in red.

The secondary structure of RNA RyhB was first predicted in *E. coli* in 2002 [19]. Although RyhB-1 and RyhB-2 in *S.* Typhimurium LT2, respectively, share ~ 82% and ~ 70% sequence identity with *E*. *coli* RyhB [27], the secondary structure of RyhB-1 and RyhB-2 in *Salmonella* has not been described to date. To better guide future research on *Salmonella* RyhB-1 and RyhB-2, we predicted their secondary structure using RNAstructure. The results predict that RyhB-1 in SE50336 is folded into three-stem loop structures that are similar to the predicted structure of RyhB in *E. coli* (Figure 1A). Although the two RyhB paralogs contain a highly conserved 33-bp core region (5′-ACGACATTGCTCACATTGCTTCCAGTATTATTT-3′) (Figure 1, red base) and consensus Fur (ferric uptake regulator) binding sites [29], the predicted structure of RyhB-2 had a big stem loop that differed from that of RyhB-1 (Figure 1B).

### RyhB-1 controls *Salmonella* growth under the condition of hypoxia and iron deficiency and in SIE

**Figure 2:**
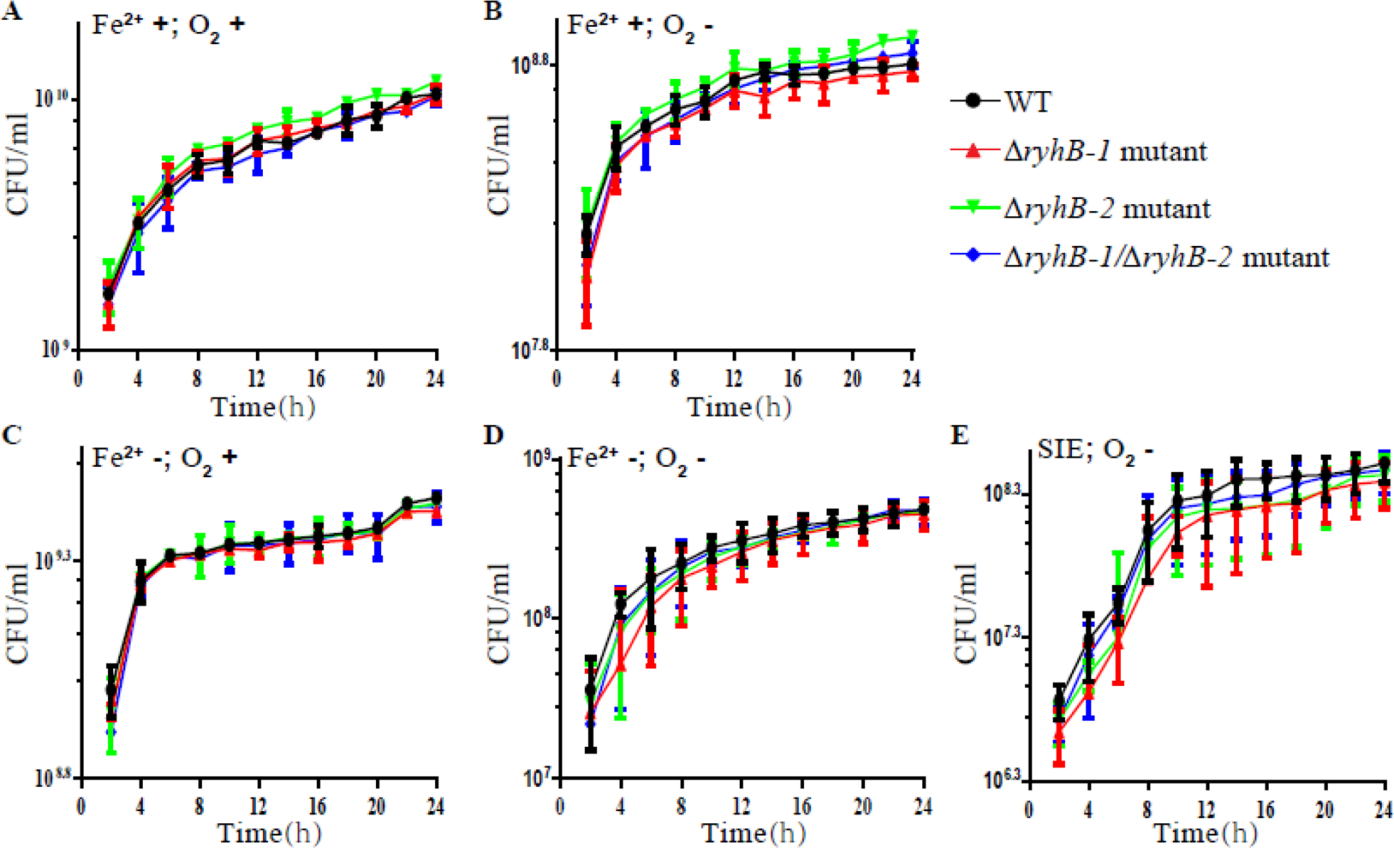
**Growth curves of S. Enteritidis 50336 WT, △ryhB-1 mutant, △ryhB-2 mutant, and △ryhB-1/△ryhB-2 mutant in different culture conditions**. The number of colony-forming unit per millilitre (CFU/mL) of triplicate cultures were determined at a 2 h interval. Data are the means of three independent experiments. Statistical analysis was performed using unpaired Student’s *t*-test. Culture conditions of SE50336 WT and all mutants are as follows: **A** LB broth aerobically with shaking at 160 rpm at 37 °C; **B** iron-limited LB broth aerobically at 37 °C; **C** LB broth anaerobically with shaking at 160 rpm at 37 °C; **D** iron-limited LB broth anaerobically at 37 °C; and **E** simulated intestinal environment anaerobically at 37 °C.

To explore the effects of RyhB on the growth of SE50336, we constructed an *ryhB-1* deletion mutant (△*ryhB-1*), an *ryhB-2* deletion mutant (△*ryhB-2*), and a double deletion mutant (△*ryhB-1*/△*ryhB-2*) and determined their growth curves in comparison with the SE50336 wild-type strain (WT) under the following conditions: LB broth aerobically with shaking at 160 rpm, LB broth anaerobically, iron-deficient LB broth aerobically with sharking at 160 rpm, iron-deficient LB broth anaerobically, and SIE. The results showed that the growth of mutants △*ryhB-1*, △*ryhB-2*, and △*ryhB-1*/△*ryhB-2* was similar to that of the WT with no significant difference (*p* > 0.05) when cultured in LB broth aerobically with shaking at 160 rpm, LB broth anaerobically, or iron-deficient LB broth aerobically with shaking at 160 rpm (Figure 2A–C). This indicated that RyhB-1 and RyhB-2 were not involved in the response to a single stress condition: hypoxia or iron-deficiency. However, when cultured in iron-deficient LB broth anaerobically, the growth rate of the △*ryhB-1* mutant was significantly slower than that of WT at 4 h (*p* < 0.05), whereas the △*ryhB-2* and △*ryhB-1*/△*ryhB-2* mutants showed similar growth with WT (Figure 2D). This suggested that RyhB-1 but not RyhB-2 affected *S*. Enteritidis growth in the exponential phase when it encountered both hypoxia and iron-deficiency stress. Compared with the WT strain, growth of the △*ryhB-1* mutant was significantly decreased in both the log and stationary phases when cultured in SIE (*p* < 0.01), while growth of the △*ryhB-2* mutant only experienced decrease from 6 to 24 h (*p* < 0.05) and the growth of the △*ryhB-1*/△*ryhB-2* mutant was decreased from 10 to 18 h (*p* < 0.05) (Figure 2E). This indicated that deletion of *ryhB-1* led to an obvious growth defect in SIE. Together, these results suggest that RyhB-1 plays an important role in response to nutrient and iron deficiency stress and hypoxia stress, while RyhB-2 has little effect on *S*. Enteritidis growth under the conditions examined in this study.

### Hypoxia, iron deficiency, and SIE upregulate RyhBs transcription

**Figure 3:**
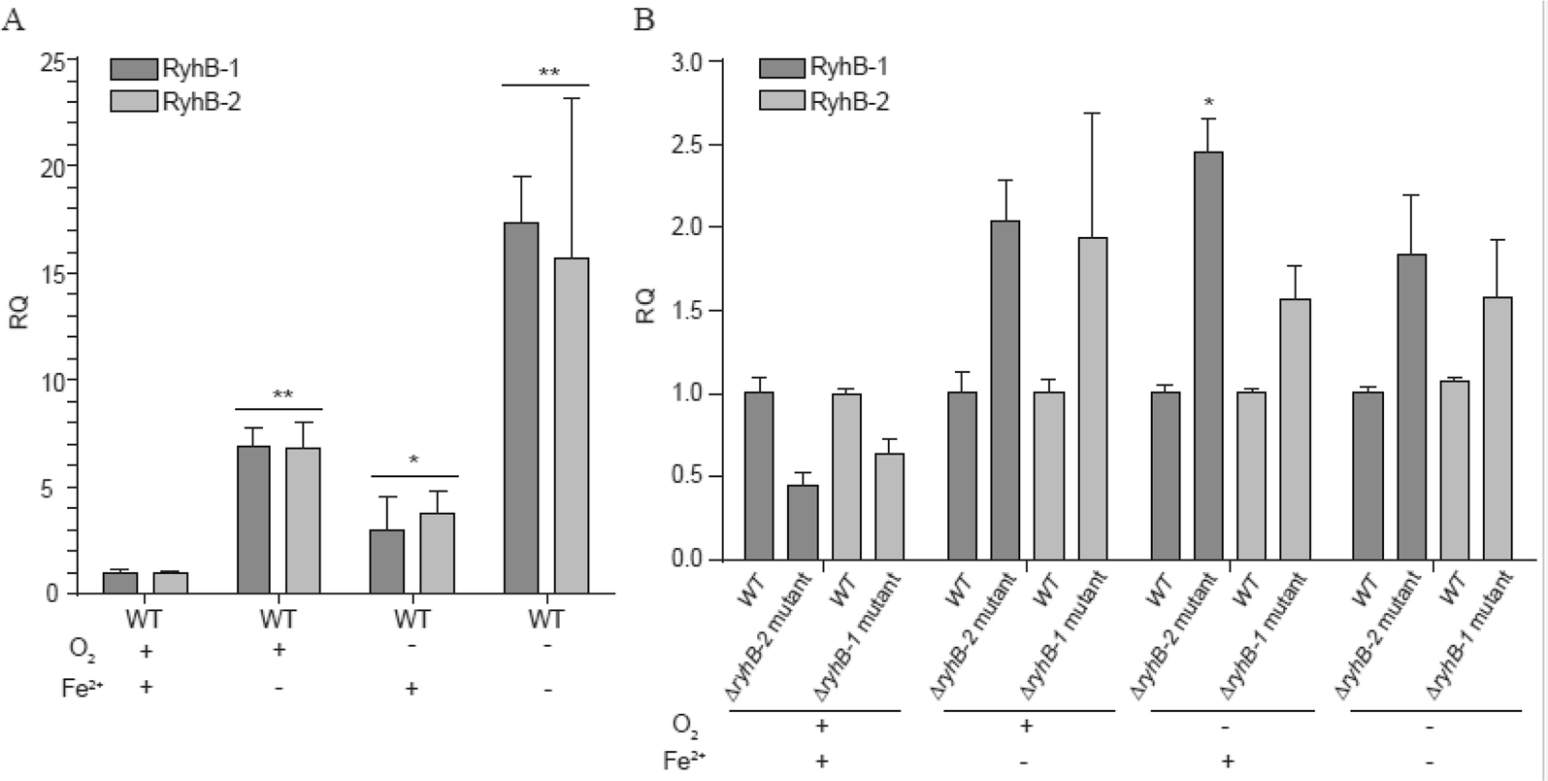
**Expression levels of RyhB-1 and RyhB-2 under anaerobic or/and iron-limited conditions, and SIE conditions**. **A** Expression levels of RyhB-1 and RyhB-2 in WT when cultured to stationary phase in anaerobic or/and iron-limited conditions compared with that cultured in LB broth aerobically. **B** Expression levels of RyhB-1 and RyhB-2 in △*ryhB-2* mutant or △*ryhB-1* mutant under anaerobic or/and iron-limited conditions compared with WT that are cultured in LB broth aerobically. Under each of the culture conditions, the expression levels of RyhB-1 and RyhB-2 in WT were used as the baseline and the expression value is defined as 1. RQ means relative quantification values of RyhB-1/RyhB-2 expression in the mutants in relative to that in WT. All assays were performed in triplicate. *p < 0.05, **p < 0.01, one-way ANOVA.

The expression of RyhB in other bacteria could be induced by sensing environmental signals such as iron limitation and exposure to hydrogen peroxide [19, 29]. To investigate the transcription levels of RyhB-1 and RyhB-2 in SE50336 under hypoxia, iron deficiency, and SIE conditions, we determined the expression of these two sRNAs by qRT-PCR. Compared with RyhB expression when the SE50336 WT strain was cultured in LB broth aerobically to stationary phase, the transcription levels of both RyhB-1 and RyhB-2 were distinctly induced by three-fold (*p* < 0.05) and seven-fold (*p* < 0.01) under conditions of hypoxia and iron deficiency, respectively. However, the most distinct induction was more than 15-fold (*p* < 0.01) under iron limitation with anaerobic conditions (Figure 3A). These findings indicated that iron limitation and hypoxia induced the expression of both RyhB-1 and RyhB-2. In addition, the transcription of one RyhB slightly decreased in another RyhB paralog deletion strain compared to WT under aerobic culture conditions in LB broth, whereas RyhB transcription increased by almost two-fold under hypoxia or (and) low-iron conditions in LB broth (Figure 3B). This suggests the existence of a complementary relationship between RyhB-1 and RyhB-2 in responding to iron limitation and hypoxia stress, i.e., one *ryhB* deletion could cause an increase in the transcription of another *ryhB* homolog to compensate for the functional defects.

**Figure 4:**
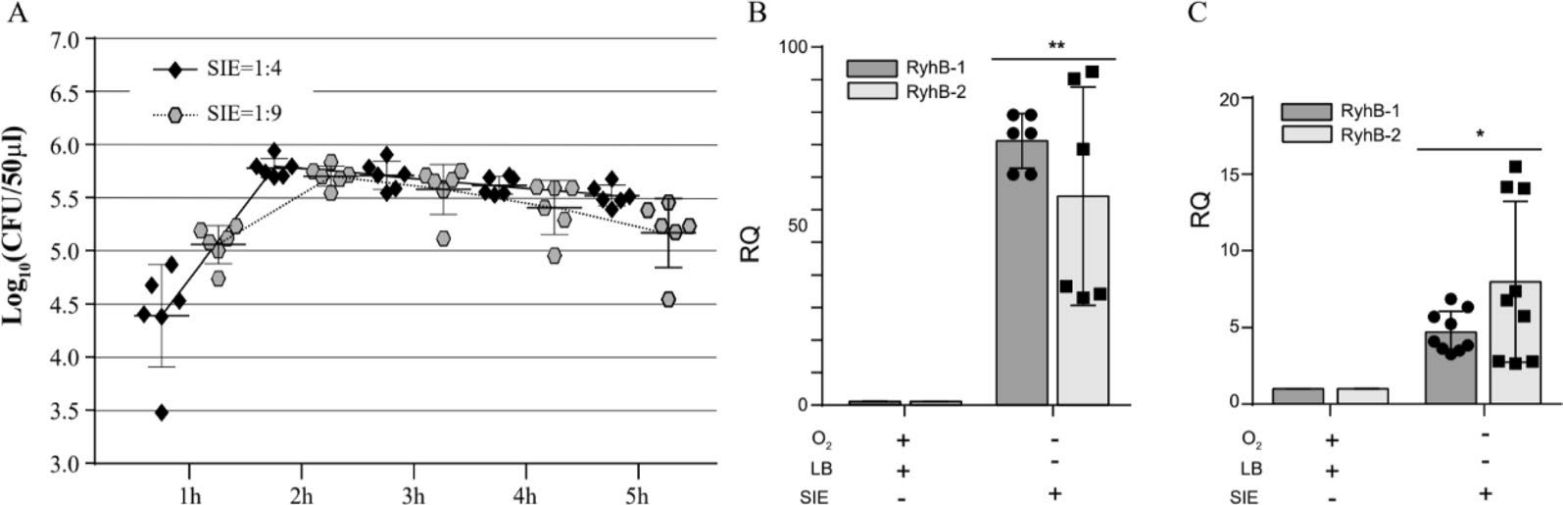
**Growth characteristics and expression levels of RyhB-1 and RyhB-2 in SE50336 when cultured in SIE**. **A** Growth characteristics of SE50336 in SIE. “SIE = 1:4 and SIE = 1:9” indicate the inoculation ratios of WT seed solution to SIE medium are 1:4 and 1:9, respectively. **B** Expression level of RyhB-1 and RyhB-2 in WT after culturing for 2 h in SIE. **C** Expression levels of RyhB-1 and RyhB-2 in WT after culturing for 3 h in SIE. RQ indicate relative quantification values of RyhB-1 and RyhB-2 expression in WT under SIE culture condition versus under LB broth aerobic conditions. All assays were performed in triplicate. *p < 0.05, **p < 0.01, one-way ANOVA.

Simulation of the intestinal environment in vitro can be used to mimic the natural infection state by *Salmonella* and study the function of RyhB in this environment. We measured the effects of different inoculation ratios on SE50336 in SIE by enumerating CFU (colony formation unit). The results showed that, with an initial inoculation ratio of 1:4 or 1:9, the CFU numbers reached to plateau between 2 and 3 h of incubation (Figure 4A). There was no difference in bacterial growth rates with different inoculation ratios, but bacterial number with an inoculation ratio of 1:4 was higher than that with an inoculation ratio of 1:9. Thus, thus the 1:4 ratio was used to prepare bacteria for qRT-PCR and subsequent RNA-Seq analysis. To investigate whether the expression of RyhB-1 and RyhB-2 was induced under SIE conditions, we detected the expression of two RyhBs in the SE50336 WT strain after incubating for 2 h or 3 h in SIE. The results showed that expression of *ryhB-1* and *ryhB-2* increased by more than 70- and 60-fold, respectively, after incubating for 2 h in SIE compared to that under aerobic conditions in LB broth (Figure 4B), whereas their expression increased by 5- and 7-fold, respectively, after incubating for 3 h in SIE (Figure 4C). The results indicated that the expression of both sRNAs could be induced by iron stress and nutrition and oxygen deprivation.

### RyhB-1 and RyhB-2 upregulate *sipA* and *sopE* expression under SIE condition in vitro

**Figure 5:**
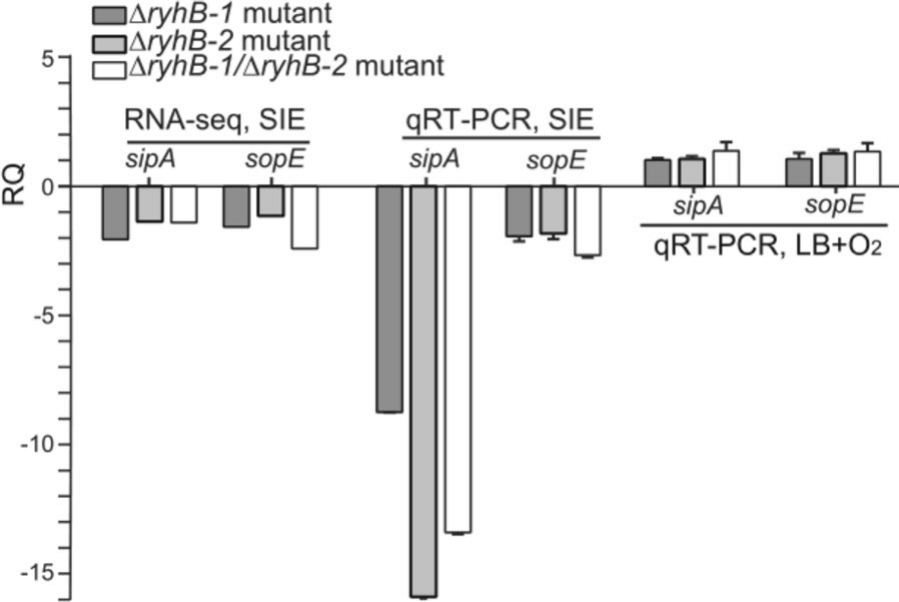
**Comparison of expression levels of sipA and sopE by RNA-seq and qRT-PCR when S. Enteritidis 50336 WT, △ryhB-1 mutant, △ryhB-2 mutant, and △ryhB-1/△ryhB-2 mutant were cultured in SIE or LB medium aerobically**. RQ represents relative expression values of *sipA* and *sopE* in △*ryhB-1* mutant, △*ryhB-2* mutant, or △*ryhB-1/*△*ryhB-2* mutant in relative to those in WT. Because the expression of *sipA* and *sopE* in WT was used as the baseline and the expression value was defined as 1, WT was not shown in the figure.

Invasion of host intestinal epithelium is a key step for *Salmonella* infection. Pathogenicity island 1 effector protein SipA and guanine nucleotide exchange factor SopE play an important role in invasion [43, 44]. Under SIE conditions, transcriptomic analysis of WT, △*ryhB-1*, △*ryhB-2*, and △*ryhB-1/*△*ryhB-2* strains showed that the deletion of *ryhB-1* and/or *ryhB-2* downregulated *sipA* and *sopE* transcription (Figure 5). To further investigate the expression pattern of *sipA* and *sopE* as influenced by RyhB-1 and RyhB-2, we assessed the expression of *sipA* and *sopE* in the WT strain and mutants △*ryhB-1*, △*ryhB-2*, and △*ryhB-1/*△*ryhB-2* by qRT-PCR when cultured in SIE or LB medium aerobically. Under SIE conditions, although the gene expression profiles obtained by RNA-Seq and qRT-PCR analyses were comparable, the relative expression fold-changes of *sipA* as detected by qRT-PCR in mutants △*ryhB-1*, △*ryhB-2*, and △*ryhB-1/*△*ryhB-2*, respectively, decreased by 8-, 16-, and 14-fold compared to the WT strain, which were significantly lower than the results detected using RNA-Seq. However, the relative expression fold-changes of *sopE* in the above mutants detected by qRT-PCR were similar to that obtained by RNA-Seq and decreased by two-fold compared to the WT strain (Figure 5). This indicated that both RyhB-1 and RyhB-2 could upregulate the expression of *sipA* and *sopE* in SIE in vitro. However, RyhB-1 and RyhB-2 did not affect *sipA* and *sopE* expression in LB broth aerobically because there was no difference in the *sipA* and *sopE* transcription between the WT and mutant strains (Figure 5). We noticed that the transcription of both RyhB-1 and RyhB-2 was inherently low when SE50336 was cultured in LB broth aerobically, which could explain why the deletion of RyhB-1 and/or RyhB-2 had little effect on *sipA* and *sopE* expression in LB broth aerobically. When SE50336 was cultured under SIE conditions, RyhB-1 and RyhB-2 transcription were drastically induced, which explains why deletion of RyhB-1 and/or RyhB-2 led to significant changes in the expression of *sipA* and *sopE*.

### RyhB paralogs directly upregulate *sipA* expression by interacting with its 5′ UTR

**Figure 6:**
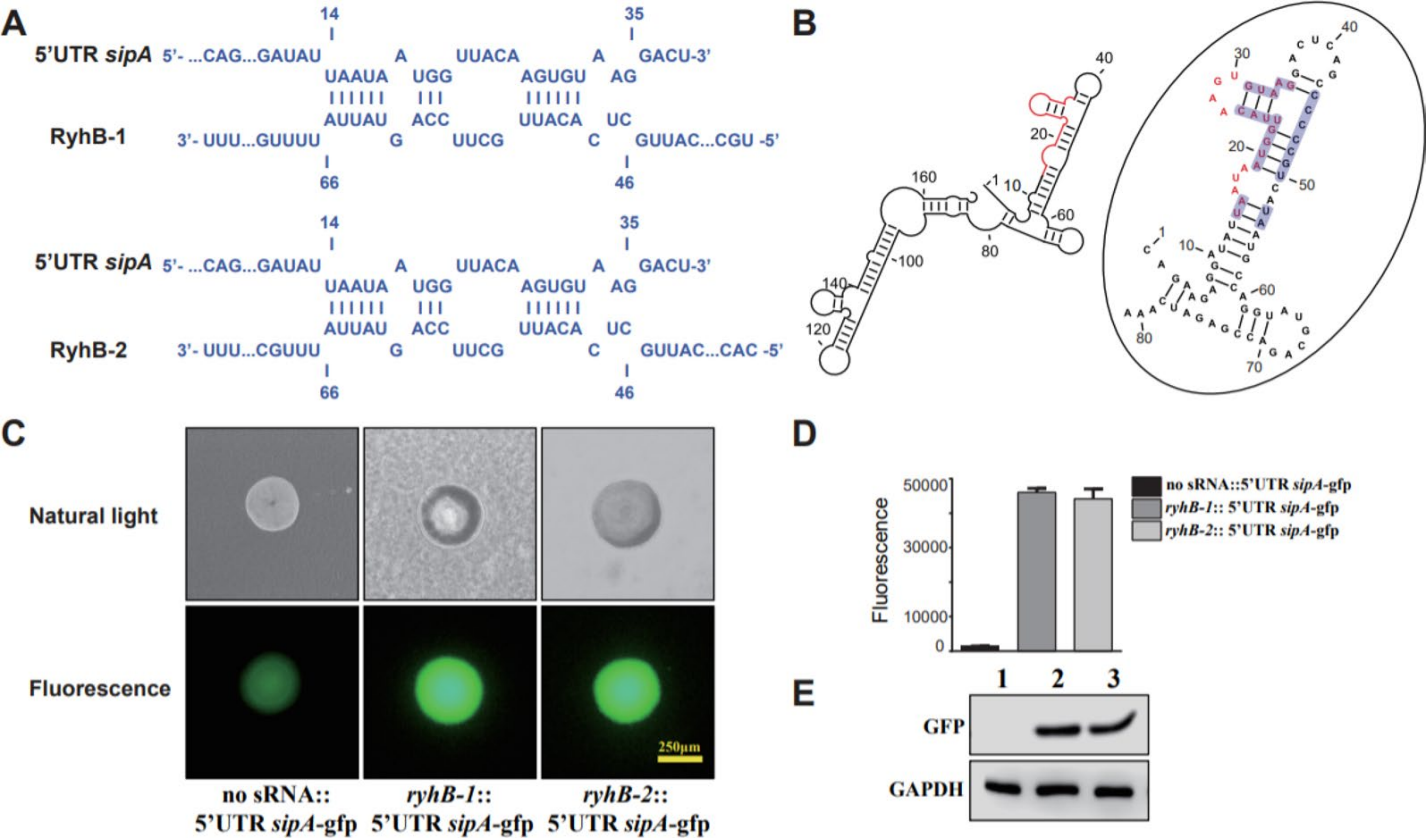
**Regulation of sipA by RyhB paralogs as determined by interaction site prediction and fluorescence assay**. **A** Interaction site prediction between RyhB-1/RyhB-2 and *sipA*. The fragment containing nt 14–35 in the *sipA* mRNA is predicted to form base pairs with RyhB-1 and RyhB-2 (nt 46–66). **B** Prediction of the *sipA* mRNA secondary structure by RNAstructure module of CLC Main Workbench (5.5) software. The 5′ UTR of *sipA* that form incomplete base pairing with RyhBs are marked in red, while the bases of the coding region that form the stem-loop structure with 5′ UTR of *sipA* are marked in blue. **C** Single colonies on LB plates imaged using the natural light mode and fluorescence mode, respectively, by an inverted fluorescence microscope. **D** Bacterial fluorescence units when cultured in LB liquid medium. The dada were analyzed statistically using unpaired Student’s *t*-test. **E** GFP protein expression in *E. coli* TOP10 containing various fusion plasmids as determined by Western blotting. 1. no sRNA::5′ UTR *sipA*-gfp; 2. *ryhB-1*::5′ UTR *sipA*-gfp; 3. *ryhB-2*::5′ UTR *sipA*-gfp. GAPDH was used as a loading control.

The result of RyhB-*sipA* interaction site prediction suggested that a region (nt 46–66) in RyhB-1 and RyhB-2, which is located in the highly conserved 33-bp region between the two sRNAs, could form base pairs with 5′ UTR of *sipA* and the first 15 bases in the coding region (nt 14–35 in 168-nucleotide sequence) (Figure 6A). In addition, we analysed the secondary structure of the 5′ UTR and the first 150 bases of the *sipA* mRNA by using RNAstructure. The result indicated that the 5′ UTR and the first 40 bases of the *sipA* coding sequence formed stem-loop structures. In particular, there is a long stem loop (Figure 6B) that possibly inhibits the binding of ribosomes to the Shine-Dalgarno (SD) sequence and decreases the translation efficiency of SipA. Combined with the results of interaction site prediction between sRNA and target mRNA, we hypothesized that RyhB paralogs bind to the 5′ UTR of *sipA*, prevent the formation of stem-loop structures, and then promote the translation of SipA. Indeed, the GFP fusion experiment showed that the fluorescence intensity of the strains carrying *ryhB-1*::5′ UTR *sipA*-gfp or *ryhB-2*::5′ UTR *sipA*-gfp was visually stronger than the strain with the “no sRNA::5′ UTR *sipA*-gfp” on LB agar plates (Figure 6C). In addition, liquid cultures of the above strains were measured for whole-cell fluorescence at a cell density of OD_600_ = 2.0. The result showed that the fluorescence units of the strains harbouring *ryhB-1*::5′ UTR *sipA*-gfp and *ryhB-2*::5′ UTR *sipA*-gfp were 32- and 31-fold higher than that of “no sRNA::5′ UTR *sipA*-gfp”, respectively (Figure 6D). The direct detection of GFP protein expression also revealed vast differences in the GFP protein abundancy, consistent with the results of liquid culture whole-cell fluorescence measurement (Figure 6E). These results indicate that the 18-nt 5′ UTR of *sipA* plays an important role in the interaction with RyhB-1 and RyhB-2 and consequently affects the SipA-GFP fusion protein expression. Thus, the findings suggest that RyhB-1 and RyhB-2 directly upregulate the expression of SipA by interacting with the 5′ UTR of *sipA*.

### *SopE* is upregulated by RyhBs

**Figure 7:**
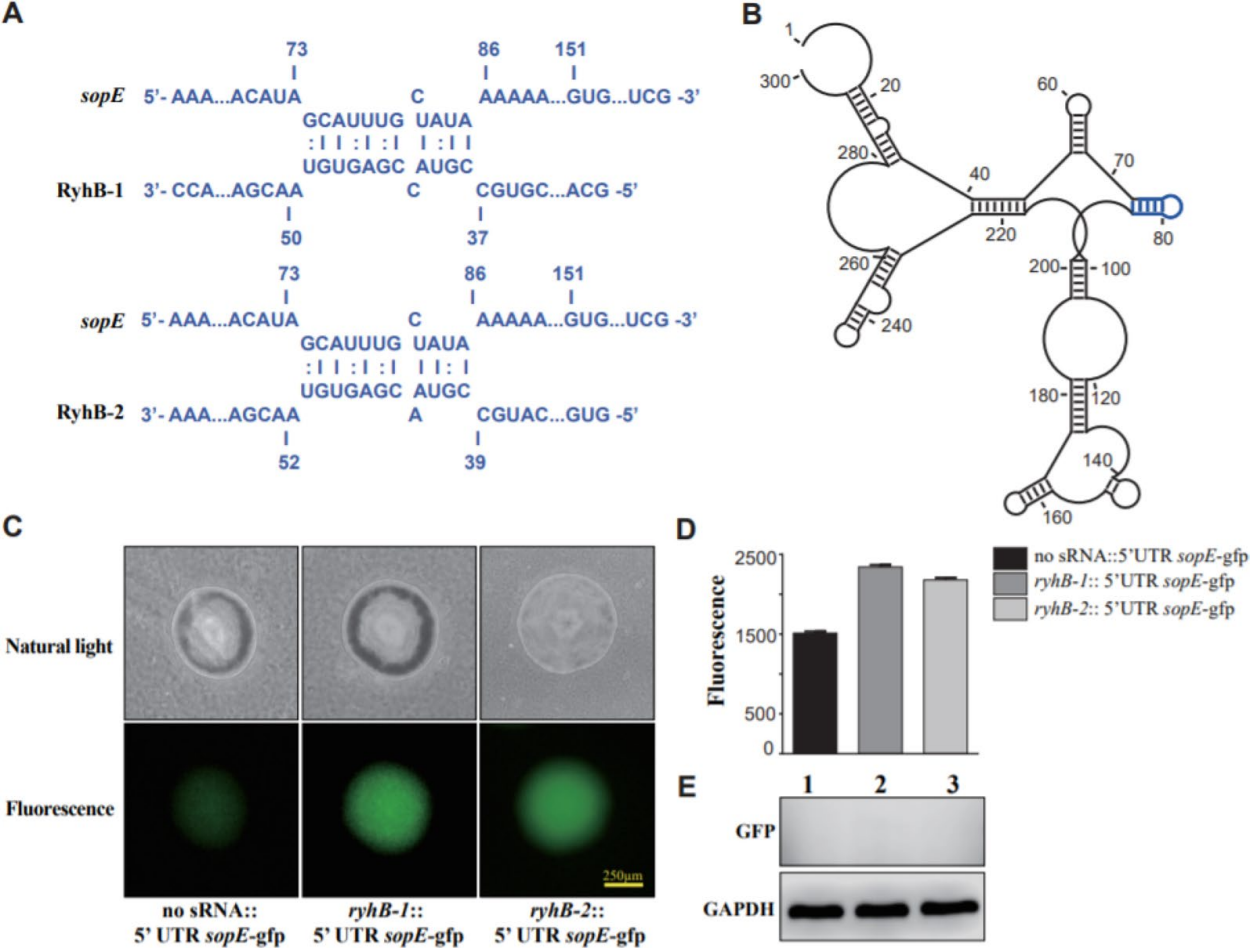
**Regulation of sopE by RyhB paralogs as determined by interaction site prediction and fluorescence assay**. **A** Interaction site prediction between RyhB-1/RyhB-2 and *sopE*. 5′ UTR of *sopE* (nt 73–86) is predicted to form incomplete base pairing with RyhB-1 (nt 37–50) or RyhB-2 (nt 39–52). **B** prediction of *sopE* mRNA secondary structure by RNAstructure module of CLC Main Workbench(5.5) software. A hairpin loop is formed in 5′ UTR of *sopE* (nt 73–86) (marked in blue). **C** Single colonies on LB plates imaged using the natural light mode and fluorescence mode by an inverted fluorescence microscope. **D** Bacterial fluorescence values in LB liquid medium. E. GFP protein expression in *E. coli* TOP10 containing various fusion plasmids as determined by Western blotting. 1. no sRNA::5′ UTR *sopE*-gfp; 2. *ryhB-1*::5′ UTR *sopE*-gfp; 3. *ryhB-2*::5′ UTR *sopE*-gfp. GAPDH was used as a loading control.

Interaction site prediction between RyhBs and the *sopE* gene as well as GFP fusion experiment were also used to study the regulation of *sopE* by RyhBs. The result showed that a very similar fragment in RyhB-1 (nt 37–50) and RyhB-2 (nt 39–52) could form base pairs with the 5′ UTR of *sopE* (nt 73–86) (Figure 7A). Secondary structure prediction of the 300-nt sequence of *sopE* (150-nt 5′ UTR and the first 150-nt coding sequence) showed that the binding region formed a hairpin loop, which might be opened via interaction with RyhB-1 and RyhB-2 (Figure 7B). However, the GFP-based reporter assay showed that the fluorescence units of the strains carrying *ryhB-1*::5′ UTR *sopE*-gfp or *ryhB-2*::5′ UTR *sopE*-gfp were only about 1.5-fold higher than that of the strain with “no sRNA::5′ UTR *sopE*-gfp” (Figure 7C–E). There was also no increased expression of the GFP protein. Whether RyhB-1 and RyhB-2 directly interact with *sopE* is uncertain and requires further investigation.

### RyhBs contribute to adhesion and invasion to intestinal epithelial cell and *Salmonella* virulence

**Figure 8:**
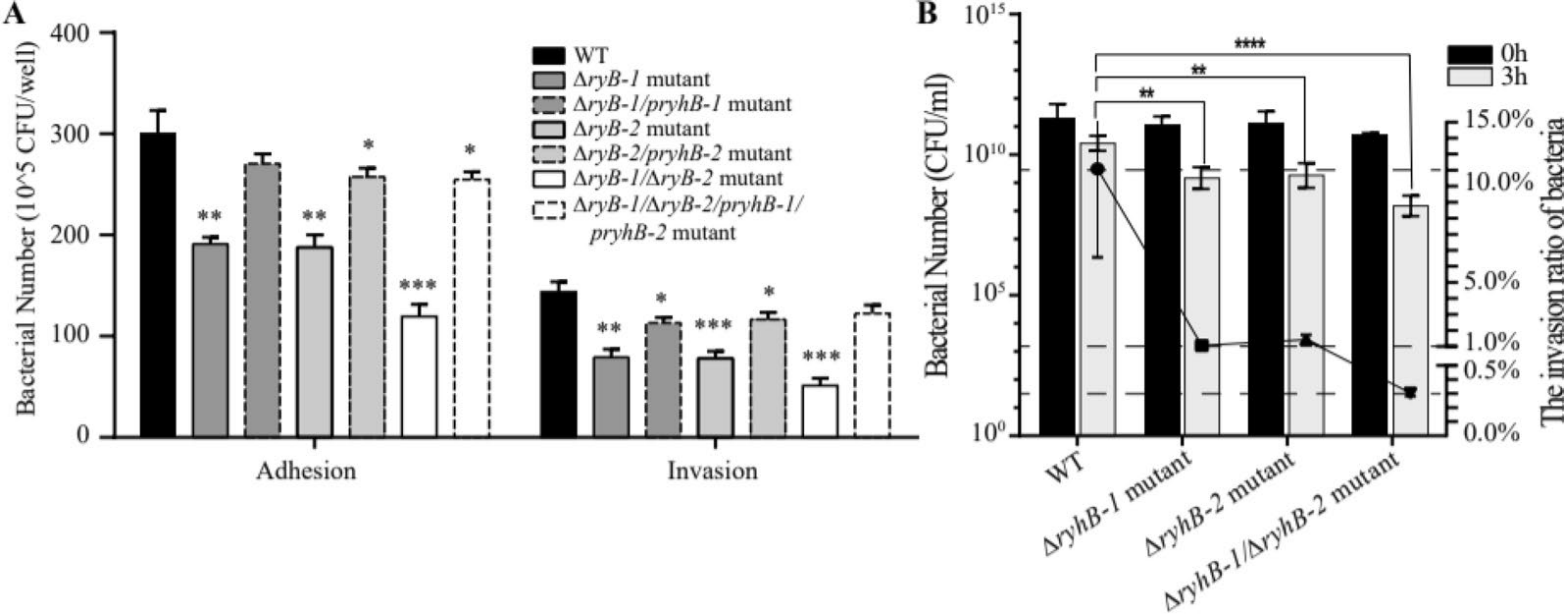
**Effects of RyhB paralogs on the adhesion and invasion of S. Enteritidis**. **A** Adherence to and invasion into Caco-2 cells by SE50336 WT, △*ryhB-1* mutant, △*ryhB-2* mutant and △*ryhB-1*/△*ryhB-2* mutant. **B** Adhesion and invasion under SIE in a MEAT model. The black and grey bars represent the numbers of WT, *ryhB-1* mutant, *ryhB-2* mutant, *ryhB-1/ryhB-2* mutant at 0 h (inoculation) and 3 h, respectively. The Y-axis on the left indicates bacterial CFUs, while the Y-axis on the right depicts the ratio of bacteria number at 3 h to the number at 0 h for each strain. Bacterial numbers are represented by bars, while the ratios are shown in a scatter plot. The comparison of invasion ratios between mutants and WT was analyzed statistically using one-way ANOVA. *p < 0.05, **p < 0.01, ***p < 0.001.

Adhesion of and invasion into epithelial cells is an important step for *Salmonella* pathogenesis. To study whether RyhBs affect this virulence trait, the WT, deletion mutants (△*ryhB-1*, △*ryhB-2*, and △*ryhB-1/*△*ryhB-2*) and complemented strains (△*ryhB-1*/*pryhB-1*, △*ryhB-2*/*pryhB-2*, and △*ryhB-1/*△*ryhB-2*/*pryhB-1*/*pryhB-2*) were used for the adhesion and invasion assays involving cultured intestinal epithelial Caco-2 cells and the MEAT model. Compared with the WT, the adhesion of single-deletion mutants △*ryhB-1* or △*ryhB-2* to Caco-2 cells was 60% of that of the WT, while the double-deletion mutant (△*ryhB-1/*△*ryhB-2*) was 35% of the WT (Figure 8A, p < 0.01). The complemented strains partially restored the adherence ability. The results of invasion into Caco-2 also showed the same trend as that of adhesion assays. Compared with the WT, the invasion level of single-deletion mutant △*ryhB-1* or △*ryhB-2* was 50% of the WT, whereas the double-deletion mutant △*ryhB-1/*△*ryhB-2* was 30% of the WT (Figure 8A, p < 0.01). The complemented strains partially restored invasion level. These findings indicated that deletion of *ryhB-1* or *ryhB-2* decreased the adhesion and invasion ability of *S.* Enteritidis, and the effect was particularly obvious with the double deletion. Thus, both RyhB-1 and RyhB-2 contribute to the adhesion and invasion of *Salmonella* into intestinal epithelial cells.

*Salmonella* invasion into epithelial cells was also detected by a MEAT model that simulates the intestinal environment. In this ex vivo model, WT and mutants strains were applied to mouse intestinal epithelia and incubated anaerobically for 3 h. The numbers of S*almonella* bacteria that adhered to and invaded into mouse intestine were determined at the end of incubation and compared with the initial inocula (time 0 h). As shown in Figure 8B, the number of WT adhering to and invading into the intestinal epithelial cells of Balb/C mice accounted for 12.08% of the original inoculum (time 0 h), whereas the adherence and invasion numbers of mutants △*ryhB-1* and △*ryhB-2* accounted for 1.07% and 1.42% of the original inocula (*p* < 0.01), respectively. Furthermore, the adhesion and invasion numbers of the double mutant △*ryhB-1/*△*ryhB-2* only accounted for 0.3% of the number of inoculations of 0 h (*p* < 0.001). These results showed that the invasion efficiencies of mutants were decreased significantly when compared with WT. In other words, the deletion of RyhB-1 and/or RyhB-2 weakened the invasion ability of *S*. Enteritidis.

## Discussion

Our study showed that there are two RyhB paralogs in SE50336 that share almost 100% sequence identity with *S.* Typhimurium RyhB. This is consistent with the findings of a previous study that described the high conservation of *ryhB* gene sequences in most *Salmonella* species [21]. The observation of highly homologous sequences was indicative of a similar function in *Salmonella*. However, due to differences in biological characteristics, particularly pathogenicity, of different salmonellae, RyhB homologs may perform various regulatory functions. Compared with RyhB-2 in SE50336, RyhB-1 has higher homology with *E. coli* RyhB. Secondary structure prediction performed in our study also demonstrated that the RyhB-1 structure in SE50336 is similar to the predicted structure of RyhB in *E*. *coli*, prompting us to infer that they similarly sense environmental stresses and regulate the expression of target genes. Nevertheless, the predicted structure of RyhB-2 differed from that of RyhB-1. RyhB-2, also named IsrE, is an island-encoded sRNA that had different expression patterns but shared the same 33-bp core region and Fur-binding sites with RyhB-1 [29]. Both similarities and differences between RyhB-1 and RyhB-2 indicated that these two homologs might similarly function in regulating the same targets as well as perform diverse functions in controlling the expression of individual targets.

Adapting to and surviving the intestinal environment is essential for *S*. Enteritidis to invade and infect its host. Here, we reported for the first time the expression patterns of two RyhB homologs under the simulated intestinal environment and under single or dual stress conditions: iron deficiency and/or hypoxia. We also measured the effects of two RyhB homologs on SE50336 growth. Under aerobic conditions in LB broth, single- and double-deletion of RyhB-1 and RyhB-2 did not affect the growth of SE50336, although their expression levels were lower than those under stress conditions. These findings indicated that the two RyhB homologs were not induced under nutrient-rich aerobic conditions. Single deletion of RyhB-1 but not RyhB-2 decreased the growth under both iron-limited and anaerobic conditions in the exponential phase, suggesting that only RyhB-1 is needed for the response to iron deficiency and hypoxia stress. Jacques et al. also showed that RyhB in *E. coli* is essential for maintaining normal growth and survival under iron starvation [45]. RyhB-1 in *Salmonella* and RyhB in *E. coli* are highly homologous in sequence and predicted structure. Based on the results in this study and published work by others, it appears that RyhB-1 but not RyhB-2 plays a major role in response to iron deficiency and hypoxia in bacteria. Interestingly, the effect of double-deletion of RyhB-1 and RyhB-2 on S. Enteritidis growth was less significant than that of single-deletion of RyhB-1 or RyhB-2. The reason of this phenomenon is unknown and needs further investigation. The expression of RyhB-1 and RyhB-2 markedly increased in the SE50336 WT strain under iron limited conditions compared to iron-rich conditions; moreover, the expression of RyhB-2 increased by two-fold in the RyhB-1 deletion mutant compared to the WT strain under iron deficiency conditions. Thus, we inferred that although RyhB-1 plays a major role in response to iron deficiency stress, RyhB-2 might compensate for RyhB-1′s function when RyhB-1 was deleted. A previous study showed that a lack of O_2_ evidently changed the expression of genes controlled by Fur and RyhB in *E. coli* K-12. RyhB targets were enriched for anaerobic respiration and metabolism processes in these conditions [46]. In our study, although the single and double deletion of RyhB-1 and RyhB-2 did not affect the growth of SE50336 under anaerobic conditions, the expression of both RyhB-1 and RyhB-2 dramatically increased under anaerobic and iron-deprived conditions, which are more similar to the environment that is encountered by *Salmonella* in the gut. Furthermore, these increases were more prominent in the simulated intestinal environment. Obviously, both RyhB-1 and RyhB-2 are involved in the regulation of target gene expression under the anaerobic and iron-deprived conditions. This is critical for SE50336 adaptation and survival in the intestine and for the development of pathogenicity. Although we tried to simulate the intestinal environment to study RyhBs expression, it is not identical to the natural intestinal environment. Expression of RyhB-1 and RyhB-2 in the real intestinal environment needs to be studied in the future. In addition, both RyhB-1 and RyhB-2 in *S.* Typhimurium are strongly induced during macrophage infection [29]. Thus, it is likely that both RyhB-1 and RyhB-2 are closely related to the pathogenicity of SE.

T3SS play a crucial role in the invasion and survival of *Salmonella* in host cells and disruption of host defenses [14]. In this study, we identified T3SS effector gene *sipA* as a target of RyhB paralogs and demonstrated that both RyhBs directly and positively regulated *sipA* expression. By combining the results of the interaction site prediction and GFP-based reporter assay, we inferred that under LB medium aerobic culture conditions with a low level of RyhBs, *sipA* transcribed and formed a stem-loop structure with its 5′ UTR, which inhibited the binding of the ribosome to the SD sequence and reduced the translation efficiency of SipA. When RyhB-1 and RyhB-2 are induced under SIE conditions, both RyhBs directly bind to the SD sequence, open the stem-loop structure, and thereby expose the ribosome binding site (RBS) and initiate translation of SipA. In *E. coli* and *Salmonella*, RyhB most commonly negatively regulates targets but sometimes positively affects target expression [24]. By far, only two targets *shiA* (permease gene of shikimate) and *cirA* (TonB-dependent transporter gene of siderophores) have been shown to be positively regulated by RyhB [47, 48]. RyhB directly base-pairs to the 5′ UTR of *shiA* to disrupt its stem-loop structure that masks the RBS, thereby allowing ribosomes to initiate *shiA* translation. This regulatory mechanism is very similar to RyhBs activating the expression of *sipA* in our study.

Although the results of qRT-PCR, and sRNA-target interaction site prediction showed that RyhBs in SE can promote the expression of *sopE*, the results of the GFP-based reporter assay indicated that the interaction between RyhBs and *sopE* might be weak. Thus, whether it is a direct or indirect interaction needs further verification. As a T3SS effector protein, the expression of *sopE* and other SPI1 T3SS is tightly regulated by its central activators HilA and HilD, which are activated by ferric uptake regulator (Fur) [49]. Meanwhile, many investigations have revealed that RyhB, the Fur-sparring partner, controls the synthesis of Fur and is also regulated by Fur [24, 50]. Thus, if interaction between RyhBs and *sopE* is indirect, the regulation of *sopE* by RyhB might be mediated by Fur. This possibility remains to be determined in further studies.

As the iron homeostasis regulators, RyhB and Fur are both involved in the pathogenicity of bacteria [24, 25]. Many investigations have revealed that RyhB contributes to bacterial virulence such as adhesion to, invasion of, and survival in eukaryotic cells. For instance, RyhB can enhance the colonization ability of uropathogenic *Escherichia coli* (UPEC) in the urethra and infectivity to mice [51]. RyhB suppresses the expression of several virulence genes, including T3SS and its secreted effectors by regulating transcriptional activator VirB in *Shigella dysenteriae* [22, 52]. RyhB participates in Fur regulon to promote the bacterial capsular polysaccharide biosynthesis and siderophore production in *Klebsiella pneumoniae* [53]. Most reports indicated that RyhB influences virulence by allowing the cell to adapt iron starvation conditions and is always mediated by Fur. However, our study first demonstrates that RyhB paralogs upregulate the expression of T3SS effector gene *sipA* and *sopE*. Meanwhile, deletion of *ryhB-1* or *ryhB-2* weakened the invasive ability of S. Enteritidis into intestinal epithelial cells, and the effect was most obvious with the deletion of both *ryhB-1* and *ryhB-2*. These results indicate that RyhB-1 and RyhB-2 play important roles in *Salmonella* invasion into epithelial cells. Furthermore, the regulatory role of RyhBs requires induction of environmental conditions such as iron deficiency, hypoxia, or mimicking the intestinal environment. Thus, it is likely that during natura infection of a host, *S*. Enteritidis RyhB-1 and RyhB-2 are induced and interact with the 5′ UTR of *sipA* and *sopE* mRNA to activate their translation, which in turn affects the ability of *S*. Enteritidis to invade epithelial cells. Despite the progress, we still don’t know whether RyhBs regulate the expression of other genes involved in the invasion, or other virulence-related genes. In addition, RyhBs control iron homeostasis, nitrate homeostasis, oxidative stress and intracellular survival in *S.* Typhimurium, *S*. Typhi or *E. coli* [23, 24]. It is not clear whether RyhBs in *S*. Enteritidis can regulate these phenotypes, and the genes associated with them. Additional studies will be necessary to further explore the regulatory role of RyhBs in pathobiology of *Salmonella* Enteritidis.

## Supplementary Information

**Additional file 1:** **Primers used for mutant construction in this study.**

**Additional file 2:** **Primers of genes for qRT-PCR.**

**Additional file 3:** **Primers used for recombinant vectors construction in this study.**

## Data Availability

The datasets used and analyzed during the current study are available from the corresponding author on reasonable request.
