# Supplementary material for: Positive regulation of Type III secretion effectors and virulence by RyhB paralogs in Salmonella enterica serovar Enteritidis
Source: Vet Res. 2021 Mar 10;52:44. doi: 10.1186/s13567-021-00915-z (PMC7944605; doi:10.1186/s13567-021-00915-z)
Supplement: Supplementary file 1 — Additional file 1: Primers used for mutant construction in this study. [file 13567_2021_915_MOESM1_ESM.docx]

**Additional file 1 Primers used for mutant construction in this study**

| **Primer** | **Sequence (5'-3')** | **Product size (bp)** |
| --- | --- | --- |
| *ryhB-1*-F | TTTGCAAAAAGAAGTAGACAAGTGCGAATGAGAATGATTATTATTGCCTTGTGTAGGCTGGAGCTGCTTCG | 1114 |
| *ryhB-1*-R | AAGCCAGGCTGAAACAAAACCCAAAAAAAAAGCCAGCAAAAGCTGGCCATATGAATATCCTCCTTAG |  |
| *vryhB-1*-F | GTTTCTCAACCAGCACAT | 761/725 |
| *vryhB-1*-R  *ryhB-2*-F  *ryhB-2*-R  *vryhB-2*-F  *vryhB-2*-R | CTCATTCCTTATCTCCTGC  CGCGGCTGAAAAAGACCATGAATTCGACATGGGATAGATA GCGGTTAGTGTGTAGGCTGGAGCTGCTTCG  ATGATAACGATTATCTTTATCAACACCGAGTAGTTGAGTTTATAACCCACATATGAATATCCTCCTTAG  ATCCATCCTCCGACTACCG  CGCTCCTCTGCCATTGTG | 1114  694/656 |
