# Supplementary material for: Positive regulation of Type III secretion effectors and virulence by RyhB paralogs in Salmonella enterica serovar Enteritidis
Source: Vet Res. 2021 Mar 10;52:44. doi: 10.1186/s13567-021-00915-z (PMC7944605; doi:10.1186/s13567-021-00915-z)
Supplement: Supplementary file 2 — Additional file 2: Primers of genes for qRT-PCR. [file 13567_2021_915_MOESM2_ESM.docx]

**Additional file 2 Primers of genes for qRT-PCR**

| Primer | Sequence (5’-3’) | |
| --- | --- | --- |
| *ryhB-1*-Fwd  *ryhB-1*-Rev | CGCGGATCCTTTCAAAGCCG  CGCGGATCCAACCTGTAGCG | |
| *ryhB-2*-Fwd  *ryhB-2*-Rev | | GCGGCTGAAAAAGACCATGA  CCTGTTCGGTACGACATTGCT |
| *sipA*-Fwd  *sipA*-Rev | | AGGCGAAGACGCTACTGATG  GCTTCGCTTCCGCTTTCTTT |
| *sopE*-Fwd  *sopE*-Rev | | AGGTAGCGCGAGTAAAGACC  CGTCATTCTTGGTTGCTCCG |
| *gyrA*-Fwd  *gyrA*-Rev | | GCATGACTTCGTCAGAACCA  GGTCTATCAGTTGCCGGAAG |
