# Supplementary material for: Positive regulation of Type III secretion effectors and virulence by RyhB paralogs in Salmonella enterica serovar Enteritidis
Source: Vet Res. 2021 Mar 10;52:44. doi: 10.1186/s13567-021-00915-z (PMC7944605; doi:10.1186/s13567-021-00915-z)
Supplement: Supplementary file 3 — Additional file 3: Primers used for recombinant vectors construction in this study. [file 13567_2021_915_MOESM3_ESM.docx]

**Additional file 3 Primers used for recombinant vectors construction in this study**

| Primer | Sequence (5’-3’) |
| --- | --- |
| pJV-300-F  pJV-300-R | gaggcatcaaataaaacgaaaggc  ctcagtatcttgttatccgc |
| *ryhB-1*-pJV-300-F  *ryhB-1*-pJV-300-R | GCGGATAACAAGATACTGAGTTCCTTATCTCCTGCAG  GCCTTTCGTTTTATTTGATGCCTCGAGACGATCCTTTTGAAG |
| *ryhB-2*-pJV-300-F  *ryhB-2*-pJV-300-R | GCGGATAACAAGATACTGAGCCTCACTTATGTCTTAACGTTAGCG  GCCTTTCGTTTTATTTGATGCCTCGTCGCCGATATTCCTGCATAAC |
| pJV-300-seq-F  pJV-300-seq-R | CCACCTGACGTCTAAGAA  CGGATTTGTCCTACTCAG |
| pXG-10SF-F  pXG-10SF-R | GCTAGCGGATCCGCTGGCT  ATGCATGTGCTCAGTATCTC |
| 5’UTR*sipA*-pXG-F  5’UTR*sipA*-pXG-R | GAGATACTGAGCACATGCATCAGAAGAGGATATTAATAATGG  AGCCAGCGGATCCGCTAGCACGCTGCATGTGCAAGCC |
| *sipA*-pXG-10SF-F  *sipA*-pXG-10SF -R | GAGATACTGAGCACATGCATATGGTTACAAGTGTAAGGACTC  AGCCAGCGGATCCGCTAGCACGCTGCATGTGCAAGCC |
| 5’UTR*sopE*-pXG-F  5’UTR*sopE*-pXG-R | GAGATACTGAGCACATGCATCAGAACAGCAAGGCTCCT  AGCCAGCGGATCCGCTAGCGGGAGTGTTTTGGATATATTTATTAG |
| pXG-10SF-seq-F | CGCCAGATATCGACGTCT |
| pXG-10SF-seq-R | AAAGACAGGCACCTCTCC |
